# Will the global HIV response fail gay and bisexual men and other men who have sex with men?

**DOI:** 10.7448/IAS.19.1.21098

**Published:** 2016-11-21

**Authors:** George Ayala, Glenn-Milo Santos

**Affiliations:** 1 The Global Forum on MSM & HIV (MSMGF), Oakland, CA, USA; 2 Department of Community Health Systems, School of Nursing, University of California, San Francisco, CA, USA; 3 San Francisco Department of Public Health, San Francisco, CA, USA

**Keywords:** HIV, gay men, men who have sex with men, sexual health, HIV services, HIV prevention, HIV treatment, treatment continuum, community, community-led, community-based

## Abstract

**Introduction:**

Gay and bisexual men and other men who have sex with men are among the small number of groups for whom HIV remains uncontrolled worldwide. Although there have been recent and notable decreases in HIV incidence across several countries, prevalence and incidence is consistently higher or rising among men who have sex with men when compared with other groups.

**Methods:**

In 2014, MSMGF (the Global Forum on MSM & HIV) conducted its third biennial Global Men's Health and Rights Study, an international, multilingual, web-based cross-sectional survey of men who have sex with men recruited through online convenience sampling. We tested hypothesized correlates (selected *a priori*) of successfully achieving each step along the HIV prevention and treatment continuum by fitting separate generalized estimating equation models adjusted for clustering by country in multivariate analyses. All models controlled for ability to meet basic financial needs, age, healthcare coverage, having a regular provider, region and country-level income.

**Results:**

Higher provider discrimination and sexual stigma were associated with lower odds of perceived access to services, service utilization and virologic suppression. Conversely, accessing services from community-based organizations focused on lesbian, gay, bisexual and transgender people; greater engagement in gay community; and comfort with healthcare providers were associated with higher odds of achieving steps along the prevention and treatment continuum.

**Conclusions:**

To meet accelerated global HIV targets, global leaders must adopt a differentiated and bolder response, in keeping with current epidemiologic trends and community-based research. The HIV-related needs of gay and bisexual men and other men who have sex with men must be addressed openly, quickly and with sufficient resources to support evidence-based, community-led and human rights-affirming interventions at scale.

## Introduction

Gay and bisexual men, and other men who have sex with men are among the small number of remaining groups for whom HIV is uncontrolled or worsening worldwide. The inability to mount true-to-fact responses that are tailored to the sexual health needs of this community threatens to undermine gains made in reaching global HIV targets set by the Joint United Nations Program on HIV/AIDS. Moreover, in the premature and overly optimistic rush towards the “end of AIDS,” the gravity of the situation for gay and bisexual men is being ignored or downplayed.

In most parts of the world outside of Eastern and Southern Africa, HIV prevalence is less than 1% of the general adult population, whereas prevalence among men who have sex with men is well over 10% [1]. HIV epidemics in high-income countries are predominantly male and primarily driven by male-to-male sexual transmission [2]. In low- and middle-income countries, men who have sex with men are 19 times more likely to be living with HIV compared with people in the general population and represent an estimated 10% of all new infections each year [3]. Although there have been recent and notable decreases in HIV incidence across several countries characterized as having generalized epidemics, prevalence and incidence is consistently higher and rising among men who have sex with men when compared with other groups [4–7].

Homophobia and sexual stigma can limit the provision and uptake of HIV prevention, treatment and care services [8–11]. Exclusion of men who have sex with men from participating in national AIDS planning processes has resulted in national plans that omit or neglect their HIV needs, which in turn contributes to inadequately funded, inaccessible and poorly targeted programmes [9]. Same-sex sexual behaviour is still criminalized in 78 countries [12]. Criminalization of homosexuality encourages human rights abuses, violence, discrimination and stigma, which worsen health disparities for men who have sex with men and their communities [13–15].

This report aims to draw attention to ongoing challenges faced by men who have sex with men in accessing the HIV services they need. The purpose of this paper is to use data gathered from a global online survey of men who have sex with men to describe HIV prevention and treatment cascades and examine their predictors in multivariable analyses. We hypothesized that for men who have sex with men in our sample: 1) sexual stigma (homophobia) and experiences of provider stigma would be negatively associated with perceived access to and utilization of services; 2) engagement with gay community and comfort with one's healthcare provider would be positively associated with access to and utilization of services; and 3) HIV service utilization and positive health outcomes would be more likely if the service were delivered by a community-based organization that specifically focused on men who have sex with men or lesbian, gay, bisexual and transgender people (LGBT).

## Methods

### Recruitment of participants

In 2014, MSMGF (the Global Forum on MSM & HIV) conducted its third biennial Global Men's Health and Rights Study (GMHR). GMHR is a web-based cross-sectional survey of men who have sex with men recruited through online convenience sampling (e.g. via organizational networks, email listservs and websites). The aim of GMHR is to describe HIV service access and its correlates. Eligible participants identified as men, reported sexual attraction to men, were age 18 years or older and were able to complete the survey in Arabic, Chinese, English, French, Portuguese, Russian or Spanish. No geographical restrictions were applied. Ethical approval was obtained from the Western Institutional Review Board.

### Measures

Participants completed a 30-minute questionnaire including items about demographics (e.g. age, country of residence, sexual orientation, ability to meet one's basic financial needs, healthcare coverage, having a regular healthcare provider); HIV status; sexual stigma or homophobia (seven items with Likert-like responses, with greater values indicating a higher degree of stigma or homophobia, *α*=0.8534 – e.g. “In your country, how many people believe that male homosexuality is a natural expression of sexuality in men, how many people believe that male homosexuality is a perversion?”); comfort with one's healthcare provider (three items with Likert-like responses, with greater values indicating a higher degree of comfort, *α*=0.8657 – e.g. “In your country, how comfortable do you feel discussing your sexual health concerns with your healthcare provider?”); experiences of provider discrimination (five items with Likert-like responses, with greater values indicating a higher degree of discrimination, *α*=0.8703 – e.g. “In the last six months, has a healthcare provider treated you poorly because you are gay/MSM?); and engagement with the gay community (10 items with Likert-like responses, with greater values indicating a higher degree of engagement, *α*=0.7304 – e.g. “During the last six months, how often have you participated in a gay men's/MSM support group?”).

### Main outcomes

The primary outcomes in this study are access to HIV prevention and treatment services (e.g. “In your community, how accessible is free or affordable HIV testing?”) and HIV prevention and treatment service utilization. Service utilization was assessed with questions such as “When was your last HIV test? In the last six months, how frequently have you been tested for HIV?” (dichotomized as having had an HIV test in the last 12 months versus not having been tested within the last 12 months); “In the last six months, how frequently have you obtained condoms?” (dichotomized as having obtained condoms at least once versus never obtaining condoms in the past six months); “In the last six months, how frequently have you participated in HIV/risk-prevention programmes for gay men/MSM?” (dichotomized as having participated in HIV programmes three or more times versus less). Pre-exposure prophylaxis (PrEP) was assessed as lifetime use with the following question: “Have you ever taken HIV medications *before* potentially being exposed to HIV, because you thought it would reduce your chances of getting HIV?” Participants were considered to have used PrEP if they responded “yes” to this question.

Among those living with HIV, linkage to care was assessed with the following question: “When you were diagnosed, did someone help you get into HIV care?” Participants were considered to have been linked to care if they reported being linked within 12 months or sooner after their HIV diagnosis. Retention in care was assessed with the following question: “How many HIV-related healthcare visits have you had in the last six months?” Participants were considered as being retained in care if they reported having more than two visits. Viral load was assessed with the following question: “What is your current viral load?” This was recorded for the outcome of virologic suppression; participants who reported either having less than 200 copies/mL or having undetectable viral load were considered virologically suppressed.

Using the primary outcomes, MSMGF adopted an intervention-centric approach to construct the HIV prevention and treatment continuum described in this report. We used this approach to highlight low service utilization for each intervention type [16], acknowledging the following: 1) the heterogeneity of prevention needs represented among diverse groups of men who have sex with men; and 2) the complex web of interacting HIV prevention modalities [17]. The number of participants who tested for HIV and received results served as the denominator for determining steps along the cascade. On the prevention end, the constructed continuum focused on HIV-negative men who have sex with men and began with the number of men who reported obtaining condoms in the last six months. On the treatment end, the constructed continuum focused on HIV-positive men who have sex with men and began with the number of men who reported being linked to care.

### Statistical analyses

Data from the 2014 GMHR permitted MSMGF to describe and evaluate correlates of the HIV prevention and treatment continuum among survey participants. We tested hypothesized correlates (selected *a priori*) of successfully achieving each step along the prevention and treatment continuum by fitting separate generalized estimating equation models adjusted for clustering by country in multivariate analyses. All models also controlled for ability to meet basic financial needs, age, healthcare coverage, having a regular provider, region and country-level income. All analyses were conducted using Stata version 13.1 (StataCorp LP, College Station, TX, USA).

## Results and discussion

**Figure 1:**
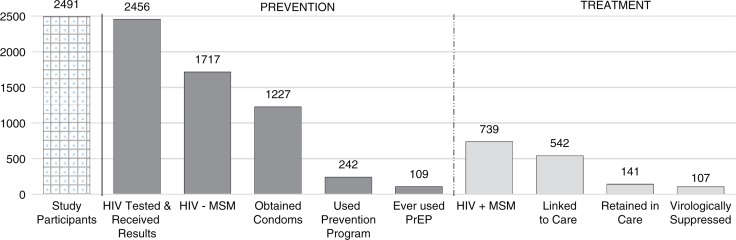
Prevention and treatment continuum among participants in the 2014 Global Men's Health and Rights Study. The prevention end of the continuum is specific to HIV-negative study participants (*n*=1717); the treatment end is specific to participants living with HIV (*n*=739); service utilization steps were reported over the 12 months prior to when the survey was taken unless otherwise noted.

A total of 4859 individuals provided consent to participate in the study and started the survey. For this analysis, we included participants with completed responses for questions of interest (*n*=2491, from 120 countries). The mean age of participants was 38 (standard deviation 12.4). Regionally, 58% of participants reported residing in Northern Europe, Western Europe or North America; 16% in Latin America; 7% in Asia; 5% in Eastern Europe or Central Asia; 5% in sub-Saharan Africa; 4% in Oceania; 2% in Central Europe; 2% in the Caribbean; and 1% in the Middle East or North Africa. Nearly all (99%) reported ever having an HIV test and receiving the test result; 30% of participants reported living with HIV (*n=*739). The analysis suggests significant gaps in the HIV prevention and treatment continuum for men who have sex with men (Figure 1). Among HIV-negative MSM (*n=*1717), 71% reported obtaining condoms in the past six months. Additionally, 73% of HIV-positive MSM reported being linked to HIV care, and 14% reported being virologically suppressed.

In the multivariable analyses focusing on the prevention end of the service continuum, participants who reported higher levels of engagement with the gay community were significantly more likely to have had an HIV test and received the result (adjusted odds ratio (aOR)=1.67, confidence interval (CI)=1.38 to 2.03); to have participated in HIV prevention programmes three or more times in the past six months (if HIV negative) (aOR=3.35, CI=2.36 to 4.75); and to have reported ever using PrEP (aOR=2.7, CI=2.0 to 3.5). Additionally, along the treatment continuum, participants who reported higher levels of engagement with the gay community were significantly more likely to be retained in care (among men living with HIV) (aOR=2.46, CI=1.22 to 4.95). These findings are aligned with other research that shows provision of safe spaces and social support and the promotion of community coherence, participation and inclusion can help reduce HIV transmission among men who have sex with men [18–20]. Community-support such as gay- and bisexual-specific health promotion can have positive impacts, such as encouraging condom use through education and non-judgmental messaging about sex and sexuality [21,22].

Comfort with one's healthcare provider was a significant predictor in the HIV prevention and treatment continuum. On the prevention end of the continuum, participants who felt more comfortable with their healthcare provider were more likely to have had an HIV test (aOR=1.22, CI=1.12 to 1.33) and to have reported ever using PrEP (aOR=1.4, CI=1.2 to 1.7). On the treatment end of the continuum, participants who felt more comfortable with their healthcare provider were more likely to be retained in care (aOR=1.18, CI=1.03 to 1.35).

Where men who have sex with men access their HIV services was also an important predictor, particularly on the prevention end of the continuum. The odds of being tested for HIV within the past 12 months (among those who had ever been tested) (aOR=1.63, CI=1.20 to 2.22) and participating in HIV prevention programmes (aOR=19.89, CI=13.42 to 29.49) were considerably higher for study participants who accessed these services from community-based organizations specifically focused on LGBT people. Previous research and normative guidance published by the United Nations Population Fund suggests that service utilization among men who have sex with men may be optimized when delivered by community-based organizations led by other gay or bisexual men [23].

In the multivariable analyses on the treatment end of the continuum, the odds of being linked to care (aOR=0.52, CI=0.31 to 0.86) and being virologically suppressed (aOR=0.44, CI=0.23 to 0.84) were significantly reduced by greater experiences of provider discrimination. Viral suppression was also negatively associated with sexual stigma (aOR=0.48, CI=0.29 to 0.82). Virologically suppressed men who have sex with men were significantly more likely to report having a regular healthcare provider (aOR=2.91, CI=1.20 to 7.07). Others in the field have also noted associations between discriminatory policies and higher HIV incidence and prevalence, limited healthcare options and reduced effectiveness of healthcare delivery [24,25]. Moreover, previous research has shown that men who have sex with men exhibit less health-seeking behaviour and greater levels of depressions, anxiety and substance misuse because of stigma [26]. Stigma and discrimination are compounded by the limited availability of sexual and reproductive health services, which increases HIV vulnerability among men who have sex with men, especially young gay and bisexual men [27,28].

## Conclusions

Our study observed steep gaps in the prevention and care continuum for HIV-negative and HIV- positive men who have sex with men. As predicted, our findings suggest that sexual stigma and higher provider discrimination are independently associated with lower odds of perceived service access, HIV service utilization and virologic suppression. Conversely, accessing HIV services from LGBT-focused community-based organizations, engagement in the gay community and comfort with healthcare providers are independently associated with higher odds of achieving steps along the HIV prevention and treatment continuum. Taken together, these results highlight the need for a bolder, more evidence-driven global response to HIV that openly acknowledges gay and bisexual men and other men who have sex with men and their sexual health needs. A more effective response must ensure unimpeded access to and scale-up of targeted testing, PrEP and treatment programmes. HIV service approaches must be developed, updated and aligned with normative guidance endorsed by UN agencies [23]. In addition, leaders in the global response should work emphatically towards the following goals:

1. *Full funding of comprehensive HIV prevention, care and treatment programmes that are competently delivered and tailored to the needs of men who have sex with men*. Funding levels should proactively: a) address the disproportionate HIV disease burden and increased HIV transmission rates among men who have sex with men; and b) support community-based and LGBT-led responses.
2. *Ensuring access to non-stigmatizing healthcare*. Healthcare workers need technical training and support to deliver high quality, evidence-informed and rights-based sexual health services for men who have sex with men.

The inclination of the global community to understate the problem of HIV among men who have sex with men is deeply troubling, especially in the context of our study's findings and epidemiologic research documenting persistently high or worsening HIV incidence. Political rhetoric often misrepresents HIV epidemiology and renders gay and bisexual men and other men who have sex with men invisible. The 2016 Political Declaration on HIV, endorsed by the United Nations, is the most recent example [29]. The Declaration strips all references to concentrated HIV epidemics occurring among gay and bisexual men and other men who have sex with men worldwide. The Declaration also fails to explicitly recognize the human rights and fundamental freedoms of men who have sex with men and the HIV-related strategies that most effectively meet their specific needs.

To meet accelerated global HIV targets, global leaders must adopt a differentiated response, in keeping with current epidemiologic trends and community-based research. The HIV- related needs of gay and bisexual men and other men who have sex with men must be addressed openly, quickly and with sufficient resources to support evidence-based, community-led and human rights-affirming interventions at scale.

## Authors' information

George Ayala, PsyD, is the executive director of MSMGF. Dr. Ayala is a clinical psychologist by training. MSMGF works to encourage equitable sexual health services for gay and bisexual men and other men who have sex with men worldwide, through its advocacy, technical support programmes and community-based research.
